# Hydrogen sulfide enhances salt tolerance through nitric oxide-mediated maintenance of ion homeostasis in barley seedling roots

**DOI:** 10.1038/srep12516

**Published:** 2015-07-27

**Authors:** Juan Chen, Wen-Hua Wang, Fei-Hua Wu, En-Ming He, Xiang Liu, Zhou-Ping Shangguan, Hai-Lei Zheng

**Affiliations:** 1 State Key Laboratory of Soil Erosion and Dryland Farming on the Loess Plateau, Northwest A&F University, Yangling, Shaanxi 712100, P.R. China; 2 Key Laboratory for Subtropical Wetland Ecosystem Research of MOE, College of the Environment and Ecology, Xiamen University, Xiamen, Fujian 361005, P.R. China; 3 Fujian Key Laboratory of Subtropical Plant Physiology and Biochemistry, Fujian Institute of Subtropical Botany; Xiamen, Fujian 361006, P.R. China; 4 College of Life and Environmental Sciences, Hangzhou Normal University, Hangzhou, Zhejiang 310036, P.R. China

## Abstract

Hydrogen sulfide (H_2_S) and nitric oxide (NO) are emerging as messenger molecules involved in the modulation of plant physiological processes. Here, we investigated a signalling network involving H_2_S and NO in salt tolerance pathway of barley. NaHS, a donor of H_2_S, at a low concentration of either 50 or 100 μM, had significant rescue effects on the 150 mM NaCl-induced inhibition of plant growth and modulated the K^+^/Na^+^ balance by decreasing the net K^+^ efflux and increasing the gene expression of an inward-rectifying potassium channel (*HvAKT1*) and a high-affinity K^+^ uptake system (*HvHAK4*). H_2_S and NO maintained the lower Na^+^ content in the cytoplast by increasing the amount of PM H^+^-ATPase, the transcriptional levels of PM H^+^-ATPase (*HvHA1*) and Na^+^/H^+^ antiporter (*HvSOS1*). H_2_S and NO modulated Na^+^ compartmentation into the vacuoles with up-regulation of the transcriptional levels of vacuolar Na^+^/H^+^ antiporter (*HvVNHX2*) and H^+^-ATPase subunit β (*HvVHA-β*) and increased in the protein expression of vacuolar Na^+^/H^+^ antiporter (NHE1). H_2_S mimicked the effect of sodium nitroprusside (SNP) by increasing NO production, whereas the function was quenched with the addition of NO scavenger. These results indicated that H_2_S increased salt tolerance by maintaining ion homeostasis, which were mediated by the NO signal.

Salinity is a major environmental stress that imposes both ionic toxicity and osmotic stress on plants, which lead to nutritional disorders, oxidative damage, and the resultant limitation of plant growth and crop yield^1^,^2^. The homeostasis of intracellular K^+^ and Na^+^ is essential for the metabolism of cells as well as for the growth and development of plants, particularly under saline conditions^3^. The K^+^/Na^+^ ratio in the cytoplasm determines the activation of enzymes, the maintenance of membrane potential, and osmotic adjustment in plants^1^,^4^.

To avoid the disruption caused by high salinity on plant growth and development, plants maintain the optimal K^+^/Na^+^ ratio and osmotic balance in the cytosol under salt stress, which are regulated by the mechanisms that prevent the excessive accumulation of Na^+^ 3,4. For example, one solution is to limit the transport of excess Na^+^ with the inhibition of non-selective cation channels (NSCCs) in the cells of plant roots^4^. Moreover, plants can extrude Na^+^ from the cytoplasm into apoplasts from the plasma membrane (PM)-located Na^+^/H^+^ antiporters (SOS1, Salt Overly Sensitive-1) or sequester Na^+^ into vacuoles from the tonoplast-located Na^+^/H^+^ antiporters (NHX1)^5^,^6^,^7^. These processes require energy derived from the electrochemical gradient with the cooperation of PM H^+^-ATPase, H^+^-pyrophosphatase (H^+^-PPase) and vacuolar membrane H^+^-ATPase (V-H^+^-ATPase)^8^. The SOS1 is a potential Na^+^ sensor and functions in the activation of Na^+^ extrusion under saline conditions^9^. The salt-induced activity of the SOS1 was reported for some crop species, e.g., rice and tomato^5^,^10^. Moreover, in the vacuolar membranes of plants, Na^+^/H^+^ antiporter activity was also detected and found to transport the Na^+^ from the cytoplasm to the vacuoles based on the electrochemical H^+^ gradient^11^. Furthermore, vacuolar Na^+^/H^+^ antiporter is widely considered to play a crucial role in maintaining a high K^+^/Na^+^ ratio in the cytoplasm by controlling the osmotic balance of the plant cell under saline conditions through the uptake of Na^+^ into the vacuoles^12^. Previous studies reported that the over-expression of *SOS1* and *NHX1* was negatively correlated with the accumulation of Na^+^ in the cytoplasm in different transgenic plants^13^,^14^,^15^. The Na^+^/H^+^ antiporter contributed to the H^+^ gradient across the PM and the vacuolar membrane^15^. Salt stress increases the activities of H^+^-pumps and up-regulates the expression of PM H^+^-ATPase and V-H^+^-ATPase^14^. Moreover, the activity of the Na^+^/H^+^ antiporter is coordinated with the activation of the V-H^+^-ATPase, which plays a key role in the sequestering of Na^+^ into the vacuoles^15^.

As we know, many plants tend to maintain a high K^+^/Na^+^ ratio instead of low Na^+^ concentration to adapt to high salinity conditions, because the Na^+^ and the K^+^ are two competitors for the same metabolic processes in the cytoplasm, such as protein synthesis, enzymatic reactions, and ribosome functions^1^,^4^. The NaCl-induced K^+^ efflux is an important response to high salinity in plants^1^,^4^. The abundance of transcripts of several K^+^ transporter genes, for example, the high-affinity K^+^ transport/K^+^ uptake transporter-type gene and the shaker K^+^ channel gene are differentially regulated by salt stress, reflects the diversity of mechanisms for the uptake of K^+^ through the roots of plants^1^. Notably, the inward-rectifying potassium channels (AKT1) are responsible for K^+^ uptake from the environment outside the root epidermis and exhibit high K^+^/Na^+^ selectivity at physiological concentrations of K^+^ and Na^+^ 16. Sentenac *et al.*^17^ reported that salt stress regulated the uptake of K^+^ by increasing the transcript abundance of the *AtAKT1* gene in *Arabidopsis* roots. Moreover, the AKT1 also indirectly participated in the SOS pathway^1^. Qi and Spalding^18^ found that the disruption of the Na^+^ efflux by a *sos* mutant resulted in excessive Na^+^ content in the cytoplasm, which inhibited the AKT1 and the growth of plant because of insufficient K^+^ uptake. Additionally, the early seedling growth and the development of an *akt1* mutant were both affected by salt, which suggested a fundamental role for the AKT1 in the maintenance of the intracellular K^+^/Na^+^ balance in NaCl-treated plants^1^,^18^.

Hydrogen sulfide (H_2_S) is a highly soluble, colourless, and inflammable gas that has long been known for the toxic effects. However, within the last decade, H_2_S has been regarded as an important molecular signal in plants. Recently, the gas was proposed as another major endogenous gasotransmitter, similar to nitric oxide (NO) and carbon monoxide (CO)^19^. H_2_S has an obvious signal regulation function in plants. For example, H_2_S promotes seed germination, ameliorates the copper-induced damage to the plasma membrane integrity of cells in wheat root tips, and decreases chlorophyll loss and alleviates oxidative damage in sweet potato seedling leaves^20^,^21^. Moreover, B^3+^ toxicity, Cr^6+^ toxicity, and Cd^2+^ toxicity in plants can also be alleviated by H_2_S^22^,^23^,^24^. Similarly, our prior study showed that H_2_S increased photosynthesis in *Spinacia oleracea* seedlings and protected barley seedlings against aluminium toxicity^25^,^26^. Notably, some evidence recently demonstrated a role for H_2_S in delaying the senescence of cut flowers and prolonging the flower vase life in herbaceous and woody plants. Additionally, H_2_S has prolonged the postharvest shelf life of strawberries and plays an antioxidative role in fruits^27^,^28^. Recent studies revealed that nitric oxide (NO) could also mediate multiple responses to environmental stimuli in plants^29^. For example, NO functions as a signal to improve the salt tolerance of plants by elevating the K^+^/Na^+^ ratio, which is dependent on the activation of both the PM H^+^-ATPase and the vacuolar H^+^-ATPase enzymes and the stimulation of the Na^+^/H^+^ antiporter^30^.

Christou *et al.*^31^ reported that H_2_S induced systemic resistance to salinity in strawberry plants through the regulation of the biosynthesis of reactive species. However, the precise mechanisms by which H_2_S controls the uptake of K^+^ and the K^+^/Na^+^ ratio dynamic equilibrium in the roots of barley remain unclear. Moreover, the relation and interaction between H_2_S and NO in the regulation of K^+^ uptake and the K^+^/Na^+^ balance remain unknown in the responses to salt stress. In this study, we investigated the correlation between H_2_S and NO in the regulation of the K^+^/Na^+^ balance and in the expression of K^+^/Na^+^ transport system in NaCl-treated barley seedling roots. Furthermore, physiological mechanisms for the roles of H_2_S and NO in improving the tolerance of plants to salt stress were proposed.

## Results

### Effects of NaCl on root length, plant height and biomass

**Figure 1:**
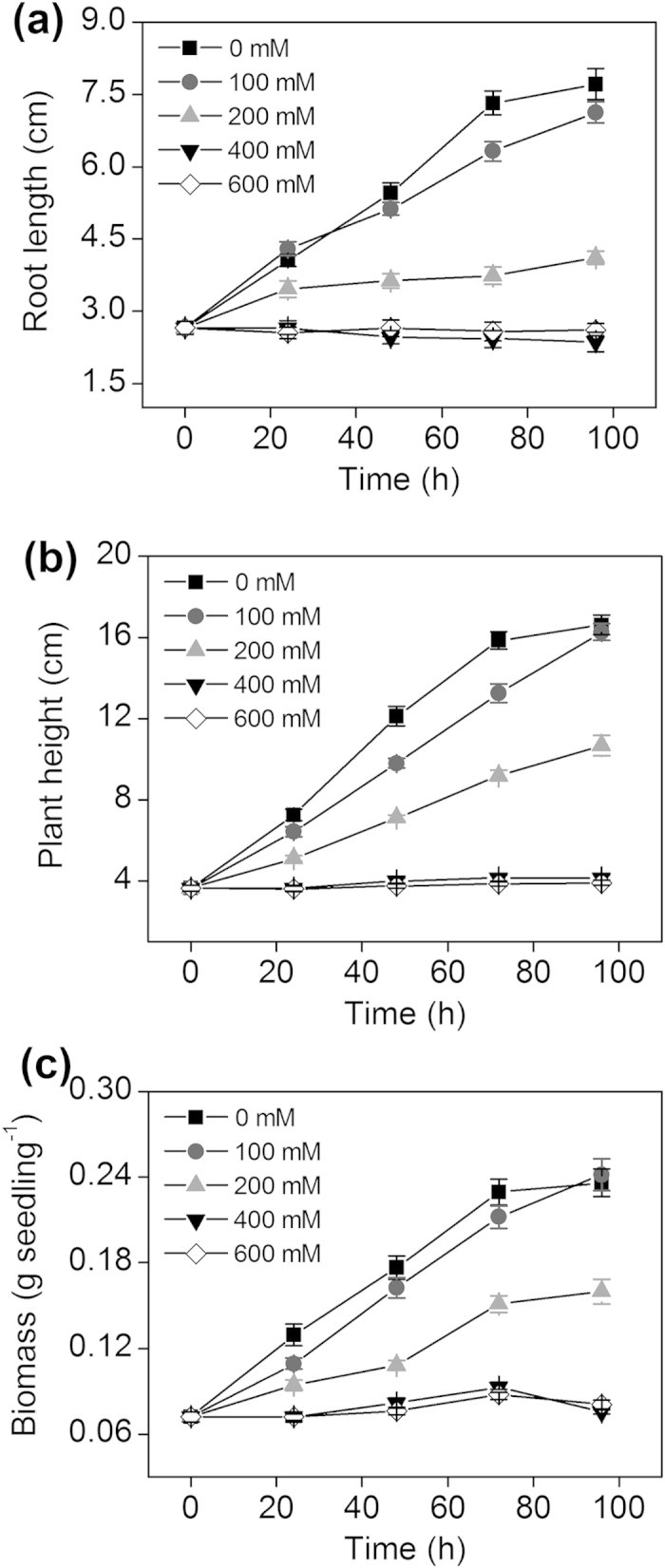
The root length (**a**), plant height (**b**), and biomass (**c**) of barley seedlings after 96 h of treatment with different concentrations of NaCl (0, 100, 200, 400 and 600 mM). Each value is the mean ± SE (*n* = 30).

The root length of barley seedlings was significantly inhibited with the increase in concentration of NaCl (Fig. 1a). The results for the plant height and biomass were consistent with those for root length in the different NaCl treatments (Fig. 1b,c). The treatment with 200 mM NaCl resulted in approximately 34%, 41% and 39% inhibition of root length, plant height and biomass in the first 48 h, respectively. However, the 100 mM NaCl treatment had no significant effect on the root length or biomass (Fig. 1a,c). Additionally, exposure to high NaCl concentrations (400 and 600 mM) for 24, 48, 72 and 96 h resulted in significant decreases in root length, plant height and biomass (Fig. 1 and Fig. 1S). Based on these results, the 150 mM NaCl treatment for 48 h was suitable and was selected for the subsequent experiment to evaluate the role of H_2_S in the amelioration of NaCl stress.

### H_2_S rescues the inhibition of NaCl on plant growth

**Figure 2:**
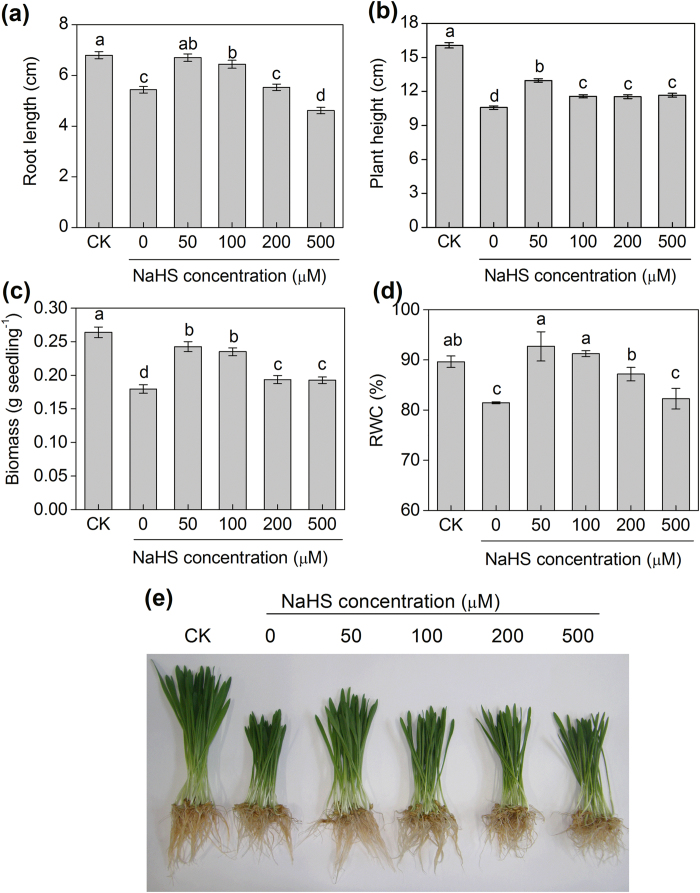
The root length (**a**), plant height (**b**), biomass (**c**), relative water content (RWC) (**d**) and symptoms (**e**) of barley seedlings treated with different concentrations of NaHS (0, 50, 100, 200 and 500 μM) and 150 mM NaCl for 48 h. CK: Hoagland solution only without adding NaHS and NaCl. Each value is the mean ± SE (*n* = 30). Columns labeled with different letters indicate significant differences with *P* < 0.05.

Low NaHS concentrations (50 or 100 μM) rescued 23% and 18% of NaCl-induced inhibition of root length compared with 150 mM NaCl alone (Fig. 2a). However, high concentrations of NaHS significantly inhibited root length (Fig. 2a). Moreover, the biomass of barley seedlings increased significantly by 35% and 31% with the low NaHS concentration (50 or 100 μM) treatments, respectively, compared with the 150 mM NaCl alone (Fig. 2c). Additionally, the 50 and 100 μM NaHS treatments significantly enhanced the RWC of leaves compared with the 150 mM NaCl alone, but there was no obvious change of the RWC in the high NaHS treatments (Fig. 2d).

### Effects of H_2_S on Na^+^ and K^+^ accumulation in leaves and roots

**Figure 3:**
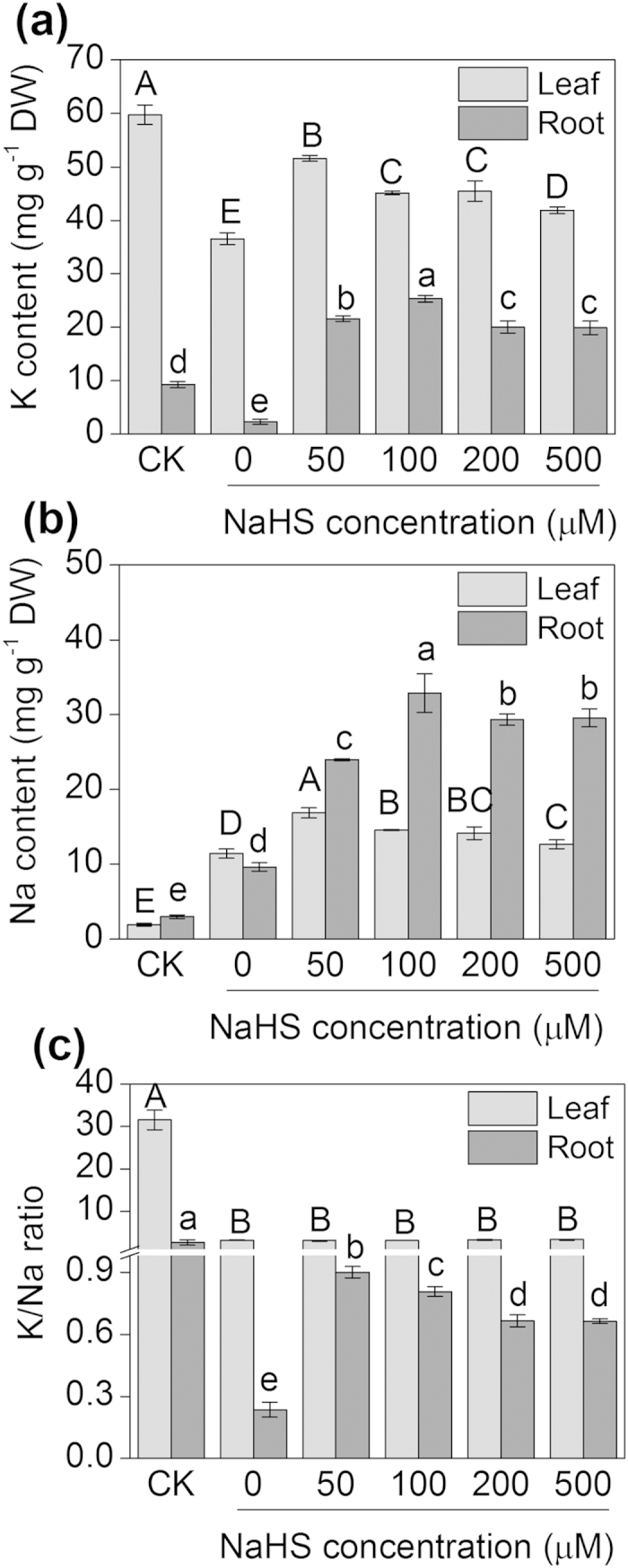
The K content (**a**), Na content (**b**), K^+^/Na^+^ ratio (**c**) in the leaves and roots of barley seedling treated with different concentrations of NaHS (0, 50, 100, 200 and 500 μM) and 150 mM NaCl for 48 h. CK: Hoagland solution only without adding NaHS and NaCl. Each value is the mean ± SE (*n* = 4). Columns labeled with different letters indicate significant differences with *P* < 0.05.

The different NaHS concentrations (50, 100, 200 or 500 μM) slightly increased the Na^+^ content and the K^+^ content to some extent, but the K^+^/Na^+^ ratio in the leaves of barley seedlings did not change compared with the solely NaCl treatment (Fig. 3). By contrast, the K^+^ and Na^+^ contents and the K^+^/Na^+^ ratios in the roots were significantly increased with the different concentration of NaHS (50, 100, 200 or 500 μM) compared with the solely NaCl treatment (Fig. 3). Moreover, the low NaHS concentrations (50 or 100 μM) were the appropriate concentrations to increase the K^+^/Na^+^ ratio in the roots of barley seedlings, which were the concentrations selected for use in the other H_2_S-related studies.

### Effect of H_2_S on Na^+^ and K^+^ fluxes in roots of barley seedlings

**Figure 4:**
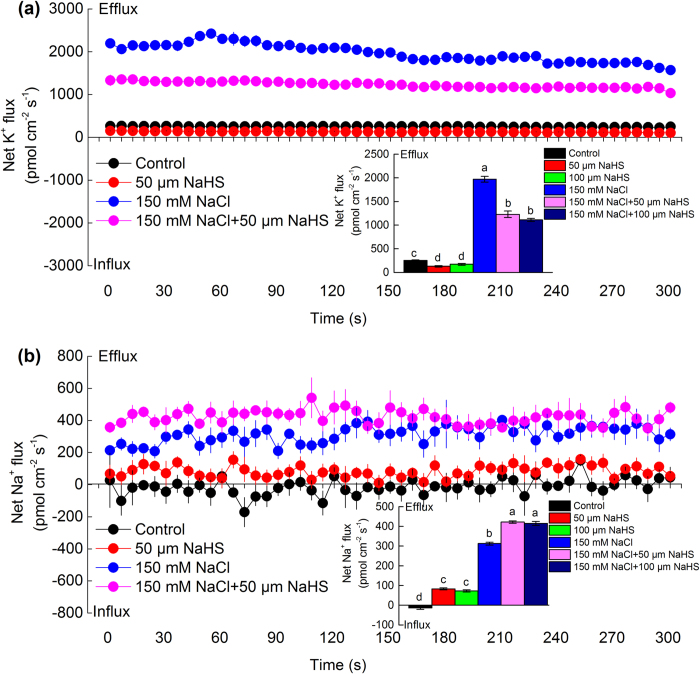
Effect of NaCl and NaHS on K^+^ and Na^+^ fluxes in the barley seedling roots. After 48 h of exposure to 150 mM NaCl, 50 or 100 μM NaHS, and 150 mM NaCl+50 or 100 μM NaHS, the net K^+^ (**a**) and Na^+^ (**b**) fluxes from the meristem root zone (100 μm from the tips) of barley seedlings. The negative value in the figures represents the net influx and positive value represents the net efflux. Each point represents the mean of six individual roots, and the bars represent the standard error (SE) of the mean. The inserted section shows the mean K^+^ and Na^+^ fluxes and SE within the measuring periods. Columns labeled with different letters indicate significant differences with *P* < 0.05.

As shown in Fig. 4a,b, the control plants exhibited a slight outward flux of K^+^ and an inward flux of Na^+^. Compared with the control plants, the 50 and 100 μM NaHS treatments slightly inhibited the efflux of K^+^ but slightly increased the efflux of Na^+^ into barley roots under normal conditions. However, after barley seedlings were treated with 150 mM NaCl for 48 h, the rectification of K^+^ flux in the root tips exhibited the evident efflux and reached the maximal mean K^+^ efflux of 1 970 pmol cm^−2^ s^−1^ (Fig. 4a). It is noted that the K^+^ efflux in the salt-treated barley seedling roots was significantly reduced by 38% and 44% under 50 or 100 μM NaHS, respectively (Fig. 4a). In contrast to the results for the net K^+^ flux, the net Na^+^ efflux in the salt-treated barley seedling roots was significantly elevated to a mean rate of 312 pmol cm^−2^ s^−1^ (Fig. 4b). Moreover, an additional supplement of 50 or 100 μM NaHS resulted in significant increases in the net Na^+^ efflux in barley roots by 34% and 33%, respectively (Fig. 4b).

**Figure 5:**
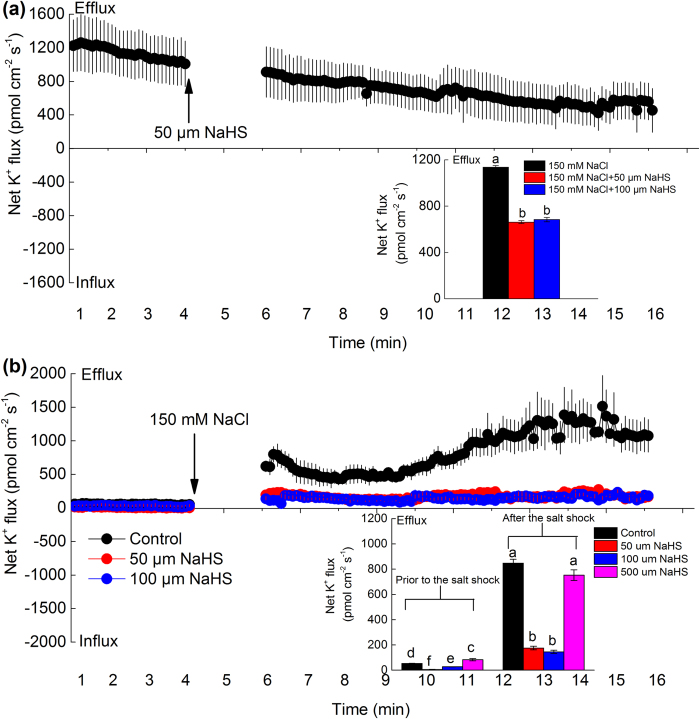
Changes in net K^+^ flux after NaHS (**a**) or NaCl (**b**) transient addition in the barley seedling roots. (**a**) The transient effect of 50 or 100 μM NaHS on K^+^ flux in 150 mM NaCl-treated barley seedling roots. Prior to NaHS addition, steady K^+^ fluxes from the meristem root zone (100 μm from the tips) of barley seedlings were examined for ~4 min. (**b**) the transient effect of 150 mM NaCl on K^+^ flux in 50 or 100 μM NaHS-pretreated barley seedlings roots. Prior to NaCl addition, steady K^+^ fluxes from the meristem root zone (100 μm from the tips) of barley seedlings were examined for ~4 min. Each point represents the mean of six individual roots and the bars represent the SE of mean. The inserts show mean K^+^ fluxes and SE within the measuring periods. Columns labeled with different letters indicate significant differences with *P* < 0.05.

To further confirm these results, the net K^+^ flux in barley seedling roots was also measured by transient additions of NaHS or NaCl. As shown in Fig. 5a, the transient application of 50 or 100 μM NaHS clearly inhibited the 150 mM NaCl-induced K^+^ efflux, which was approximately 42% and 40% lower than that without NaHS, respectively. Moreover, barley seedlings were first pre-treated with the different NaHS concentrations (50, 100 and 500 μM NaHS) for 30 min, and then transiently added 150 mM NaCl for measuring the net K^+^ flux in barley roots. These results showed that net K^+^ flux did not change obviously among the four treatments before the salt shock, but after the 150 mM NaCl shock, the K^+^ efflux reached the maximum mean of 847 pmol cm^−2^ s^−1^ (Fig. 5b). Similarly, short-term treatment with low NaHS concentrations (50 or 100 μM) also significantly reduced the K^+^ efflux, but the high NaHS concentration (500 μM) had no obvious effect compared with that of the control (the solely salt-treated seedlings) in barley roots (Fig. 5b).

### H_2_S-induced the protein expression of H^+^-ATPase and Na^+^/H^+^ antiporter is NO-dependent

**Figure 6:**
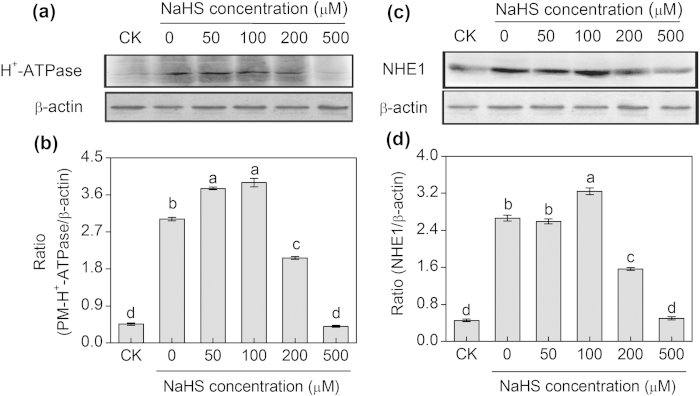
Western-blot analysis of root PM H^+^-ATPase (**a**) and vacuolar Na^+^/H^+^ antiporter (NHE1) (**c**) of barley seedlings treated with 150 mM NaCl and various NaHS concentrations (0, 50, 100, 200 and 500 μM) for 48 h. CK: Hoagland solution only without adding NaHS and NaCl. Three independent experiments were conducted, and the immunoblotting results show similar trends of protein expression. The relative protein expression level is shown as the ratio of PM H^+^-ATPase to β-actin (**b**) and NHE1 to β-actin (**d**) analyzed with Quantity One software. Mean values ± SE were calculated from independent experiments and columns labeled with different letters are significantly different at *P* < 0.05.

**Figure 7:**
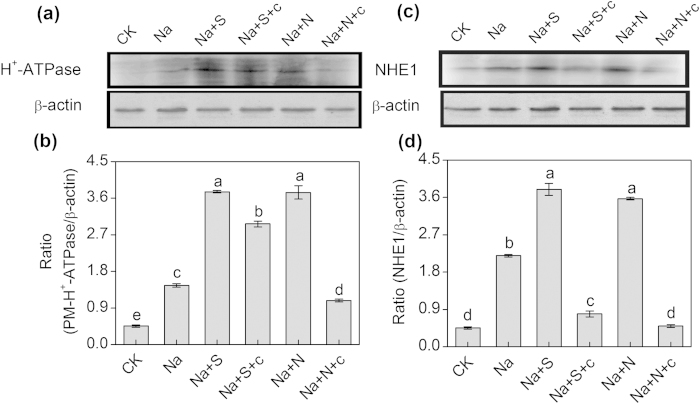
Western-blot analysis of root PM H^+^-ATPase (**a**) and vacuolar Na^+^/H^+^ antiporter (NHE1) (**c**) of barley seedlings treated with 150 mM NaCl (Na), 150 mM NaCl+100 μM NaHS (Na+S), 150 mM NaCl+100 μM NaHS+200 μM cPTIO (Na+S+c), 150 mM NaCl+100 μM SNP (Na+N), 150 mM NaCl+100 μM SNP+200 μM cPTIO (Na+N+c) for 48 h, respectively. CK: Hoagland solution only without adding NaHS and NaCl. Three independent experiments were conducted, and the immunoblotting results show similar trends of protein expression. The relative protein expression level is shown as the ratio of PM H^+^-ATPase to β-actin (**b**) and NHE1 to β-actin (**d**) analyzed with Quantity One software. Mean values ± SE were calculated from independent experiments and columns labeled with different letters are significantly different at *P* < 0.05.

As shown in Fig. 6a,b, the levels of protein expression of PM H^+^-ATPase in the 50 or 100 μM NaHS-treated seedlings reached maximum values, which increased by 25% and 30%, respectively, compared with the control (0 μM NaHS). Similarly, the level of protein expression of NHE1 in barley seedling roots clearly increased in the presence of 100 μM NaHS, which was 1.24-fold higher than that of the control (0 μM NaHS) (Fig. 6c,d). Additionally, to clarify the relationship between H_2_S and NO in response to salt stress, SNP, a donor of NO, and cPTIO, a scavenger of NO, were used in the following experiments. As shown in Fig. 7a,b, the levels of protein expression of PM H^+^-ATPase in 100 μM NaHS and in 100 μM SNP-treated seedlings reached maximum values. However, compared with the solely NaCl treatment, the increased in the protein expression of PM H^+^-ATPase induced by the NaHS and SNP was completely reversed with the addition of cPTIO. Same results were found for the protein expression of NHE1. The 100 μM NaHS and 100 μM SNP significantly increased the levels of the protein expression of NHE1, which were 1.74- and 1.63-fold higher than that of the control (the solely NaCl-treated seedlings), and these increased levels of protein expression were completely reduced by the addition of cPTIO (Fig. 7c,d).

### Effect of H_2_S and NO on transcriptional levels of K^+^/Na^+^ balance-related genes

**Figure 8:**
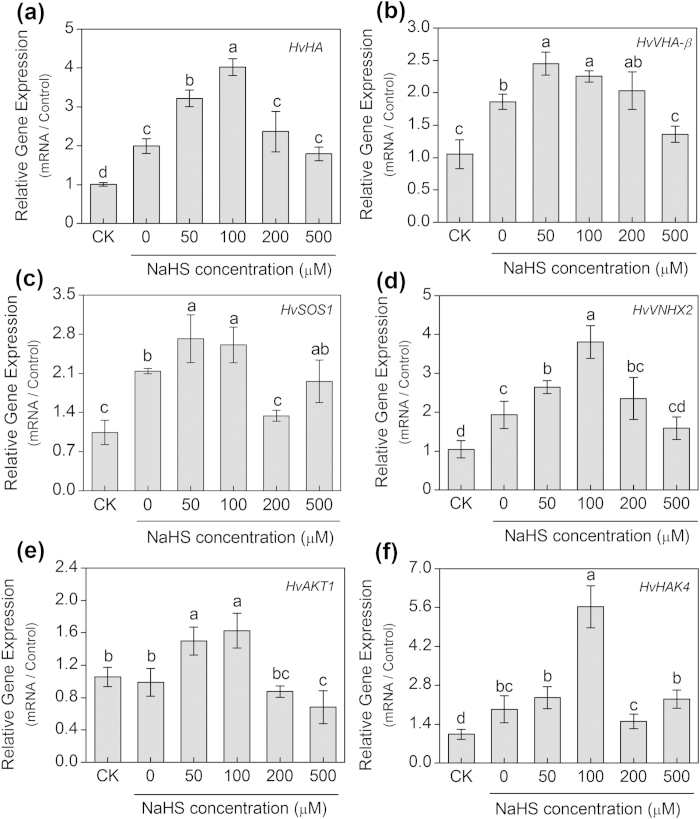
Real-time quantitative PCR analysis of relative transcript abundance of *HvHA* (a), *HvVHA-*β (b), *HvSOS1* (c), *HvVNHX2* (d), *HvAKT1* (e) and *HvHAK*4 (f) mRNA accumulation in the roots of barley seedling treated with NaCl (150 mM) and various NaHS concentrations (0, 50, 100, 200 and 500 μM) for 48 h. CK: Hoagland solution only without adding NaHS and NaCl. Transcriptional expression of the six genes was performed by normalization with a reference gene (*Hvactin*). Mean values ± SE were calculated from three independent experiments. Columns labeled with different letters indicate significant differences with *P* < 0.05.

**Figure 9:**
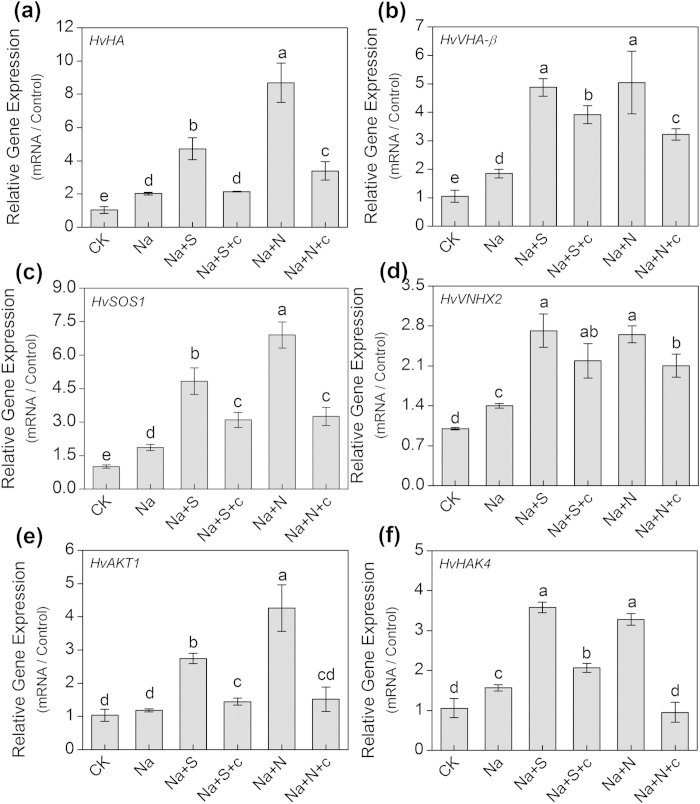
Real-time quantitative PCR analysis of relative transcript abundance of *HvHA* (a), *HvVHA-*β (b), *HvSOS1* (c), *HvVNHX2* (d), *HvAKT1* (e) and *HvHAK4* (f) mRNA accumulation in the roots of barley seedling treated with 150 mM NaCl (Na), 150 mM NaCl+100 μM NaHS (Na+S), 150 mM NaCl+100 μM NaHS+200 μM cPTIO (Na+S+c), 150 mM NaCl+100 μM SNP (Na+N), 150 mM NaCl+100 μM SNP+200 μM cPTIO (Na+N+c) for 48 h, respectively. CK: Hoagland solution only without adding NaHS and NaCl. Transcriptional expression of the six genes was performed by normalization with a reference gene (*Hvactin*). Mean values ± SE were calculated from three independent experiments. Columns labeled with different letters indicate significant differences with *P* < 0.05.

Among all treatments, the transcript levels of *HvHA* and *HvVHA-β* in the 100 μM NaHS-treated seedlings were the highest, which were increased by 102% and 21%, respectively compared with the control (0 μM NaHS) (Fig. 8a,b). Similarly, the relative transcript abundance of the *HvSOS1* and *HvVNHX2* genes reached the maximum in the 100 μM NaHS-treated seedlings, which were 1.23- and 1.97-fold higher than that of controls (0 μM NaHS), respectively (Fig. 8c,d). Moreover, 100 μM NaHS remarkably up-regulated the relative transcript levels of *HvAKT1* and *HvHAK4* genes, which was 1.66- and 2.92-fold higher than those of the control (0 μM NaHS), respectively (Fig. 8e,f). Additionally, as shown in Fig. 9, 100 μM NaHS and 100 μM SNP caused a significant up-regulation of the relative expression levels of *HvHA*, *HvVHA-β*, *HvSOS1*, *HvVNHX2*, *HvAKT1* and *HvHAK4*, but the expression levels of these genes were significantly reduced by the addition of cPTIO, which reversed the effects of H_2_S and NO in barley roots.

### H_2_S induces NO production

**Figure 10:**
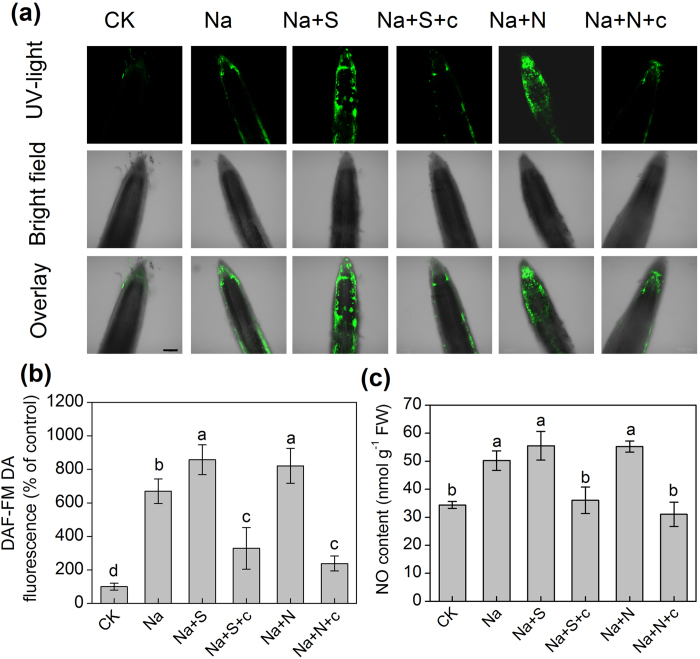
The distribution of NO in root tips by fluorescence probe DAF-FM DA detection (a,b) and H_2_S-dependent NO generation (c) of barley seedling treated with 150 mM NaCl (Na), 150 mM NaCl+100 μM NaHS (Na+S), 150 mM NaCl+100 μM NaHS+200 μM cPTIO (Na+S+c), 150 mM NaCl+100 μM SNP (Na+N), 150 mM NaCl+100 μM SNP+200 μM cPTIO (Na+N+c) for 48 h, respectively. CK: Hoagland solution only without adding NaHS and NaCl. The experiment of fluorescence (**a,b**) was repeated by six times, and similar results were obtained. Scale bar is 200 μm. Each value (**c**) is the mean ± SE (n = 3). Columns labeled with different letters indicate significant differences at *P* < 0.05.

The treatment with 150 mM NaCl for 48 h significantly increased the levels of endogenous NO, as demonstrated by a 5.7-fold increase in the fluorescence intensity compared with the controls (Fig. 10a,b). When salt-treated barley seedlings were used to measure the response to NaHS or SNP for 48 h, strong increases in the content of NO appeared in the root tips, which increased by 28% and 23%, respectively, compared with the solely salt-treated seedlings (Fig. 10a,b). Furthermore, cPTIO, a specific scavenger of NO, also inhibited the H_2_S-induced generation of NO (Fig. 10a,b). To further confirm the results from the fluorescence analysis, we determined the H_2_S-induced NO production using the Griess reagent method in barley seedling roots under salt stress. Consistently, the same tendency was found among these treatments in barley seedling roots (Fig. 10c).

## Discussion

### H_2_S attenuates salinity toxicity

As an abiotic stress, salinity affects ion homeostasis and causes ionic toxicity and osmotic stress in plants^1^,^4^,^32^,^33^. Barley is sensitive to salt stress, and the amount of Na^+^ uptake under high salinity conditions is significant^34^,^35^. In this study, with the NaCl gradient, reduced root lengths, plant heights and biomass in barley seedlings were found (Fig. 1). These findings were consistent with those that were reported in the literature. For example, after maize seedlings were grown in 100 mM NaCl for 8 d, the dry matter of shoots and roots decreased by 27.2% and 55.6%, respectively^36^. Previous studies showed that NO was involved in the physiological response to salt stress and found that NO alleviated the salt-induced inhibition of plant seedling growth^36^,^37^. Moreover, CO attenuated salinity toxicity in wheat seedlings^36^. Similar to NO and CO, H_2_S not only acts as a secondary messenger in animals but also functions in the plant kingdom^38^,^39^,^40^,^41^,^42^. However, how H_2_S functions to facilitate the plant response to stress by signalling transduction remain unclear. In this study, we investigated the effect of H_2_S on the growth response of barley to salinity toxicity. NaHS promoted a significant increase in the root length, plant height, biomass and RWC in barley seedlings under salt stress, and the low NaHS concentrations (50 or 100 μM) were the optimal concentrations to alleviate the salt-induced inhibition of plant growth in the barley seedlings (Fig. 2). These results were consistent with reports that H_2_S signalling might be required for salt tolerance in plants^31^,^43^. However, the precise mechanism by which H_2_S regulates K^+^ uptake and the K^+^/Na^+^ balance in barley roots remains unclear. Additionally, the relation and interaction of H_2_S and NO on the regulation K^+^ uptake and the K^+^/Na^+^ balance remain unknown.

### H_2_S regulates K^+^/Na^+^ balance by limiting K^+^ loss and promoting the expression of HvAKT1 and HvHAK4 in barley seedling roots

The maintenance of ion homeostasis, particularly the K^+^/Na^+^ ratio, is of critical importance for plants to accommodate to a saline environment^1^,^4^,^9^. The cytosolic K^+^ is maintained at the resting level under normal physiological conditions^44^; however, when plants sense large amounts of Na^+^, the excess Na^+^ is replaced by K^+^, which results in plant dysfunction because of the physicochemical similarities of the ions^45^,^46^. Therefore, the ability to maximize the subsequent compartmentalization into vacuoles and to minimize the net Na^+^ influx into the cytoplasm are very important to maintain a favorable K^+^/Na^+^ ratio in the cytoplasm^1^,^4^,^9^. It is likely that K^+^ also plays a vital role in the adaptation of plants to salt^47^. Previous studies reported that salt tolerance was strongly correlated with the ability of plants to retain K^+^ 7,47. For example, the barley variety with strong salt tolerance benefited from stable root cytosolic concentration of K^+^ under saline conditions^47^. Therefore, limiting the NaCl-induced leakage of K^+^ might improve the salt tolerance of the plant. H_2_S, as a signalling molecule, is involved in the resistance response of plants to high levels of salinity^31^,^43^. Lai *et al.*^48^ found that H_2_S increased the tolerance to salt by coupling the reestablishment of redox homeostasis and the increased K^+^/Na^+^ ratio in the seedlings of *Medicago sativa*. Consistent with this previous study, the application of NaHS increased the K^+^ content and the K^+^/Na^+^ ratio in the barley seedling roots in this study (Fig. 3b,c). Furthermore, the NMT study indicated that either the long-term or the transient NaHS treatments clearly induced decreases in the net K^+^ efflux from the barley roots (Figs 4a and 5a). Additionally, the long-term NaHS treatments significantly increased the net Na^+^ efflux from the root tips (Fig. 4b). To confirm these results, the barley seedlings were first pre-treated with NaHS and then NaCl was transiently added to the solution. The identical results were found, and the net K^+^ efflux significantly decreased compared with the treatment of 150 mM NaCl alone (Fig. 5b). From these results, we concluded that H_2_S played an important role in maintaining the K^+^ homeostasis and the K^+^/Na^+^ balance for barley seedling roots treated with high salinity. However, H_2_S has dual functions, either as a cytoprotectant or a cytotoxin, which depends on the concentration of H_2_S or on the status of the environment. H_2_S operates in a dosage-dependent manner either in a defensive role during osmotic stress or as a secondary messenger^21^,^49^. However, high concentrations of H_2_S might interfere with the normal growth and metabolism of plants. For example, a relatively high level of H_2_S depressed photosynthetic electron transport and impaired plant growth and development^50^,^51^.

The strong depolarization of the PM under conditions of high salinity is widely established, which causes the remarkable K^+^ loss *via* the depolarized-activated (DA) channels, e.g., the non-selective cation channels (NSCCs) and the outward rectifying K^+^ channels (KORCs) in some plants^35^,^46^. Additionally, high H^+^-ATPase activity leads to the hyperpolarization or repolarization of the PM, which limits the K^+^ efflux through the NSCCs and the KORCs^44^,^52^. In this study, the NaCl-induced transient K^+^ efflux from the root tips of barley seedlings was significantly impaired when H_2_S was applied (Figs 4a and 5a). Correspondingly, the protein and gene expression levels of the PM H^+^-ATPase were elevated by H_2_S in the barley seedlings roots (Figs 6a,b and 8a). The H_2_S-activated H^+^-ATPase might be crucial to decrease the salt-driven K^+^ efflux or to restrict the K^+^ leakage in barley seedlings under saline conditions. These results are similar to those of recent studies that suggested that the activation of PM H^+^-ATPase was required to restrict the K^+^ efflux and to retain the K^+^ in barley and *Populus euphratica*^35^,^47^.

The plants uptake of K^+^ depends on an inward-rectifying shaker K^+^ channel (AKT1) that is primarily localized in the root epidermal tissue^44^. This K^+^ channel plays an important role in the acquisition of K^+^ from the environment and is required for stable K^+^ homeostasis under salt stress^53^. The diverse transcriptional response of the *AKT1* gene to salinity is coupled to the level of plant resistance to salt. Fuchs *et al.*^54^ found that the transcriptional expression of *OsAKT1* was significantly inhibited under salt stress in *Oryza sativa*, and a similar response was found in *Arabidopsis*^53^. By contrast, salt stress greatly induced the expression level of the *AKT1* gene in *Kandelia obovata* seedling roots^55^. In the present work, we concluded that NaHS promoted the transcriptional expression of the *HvAKT1* gene in the salt-treated barley seedling roots (Fig. 8e), which suggested that H_2_S might play a crucial role in the assimilation of K^+^ with the induced the expression of the *HvAKT1* gene in the roots of the NaCl-treated barley seedlings. The high-affinity K^+^ uptake system (HAK) plays an important role in plant nutrition and is found in 5 types in barley^56^. They are ubiquitously expressed in plant tissues and function both in the plasma membrane and in the tonoplast^57^. Boscari *et al.*^58^ reported that *HvHAK4* might aid K^+^ uptake from low apoplastic K^+^ concentration conditions. In this study, the NaHS greatly induced the transcriptional expression of the *HvHAK4* gene (Fig. 8f), which suggested that H_2_S might play a vital role in facilitating K^+^ uptake by increasing the expression of the *HvHAK4* gene in barley seedling roots. Based on these results, we concluded that H_2_S mediated the homeostasis of K^+^/Na^+^ by reducing the K^+^ loss and by inducing the expression of *HvAKT1* and *HvHAK4*.

### H_2_S regulates K^+^/Na^+^ balance by activating Na^+^ extrusion in barley seedling roots

With salt stress, the maintenance of low Na^+^ content in the cytoplasm is a key factor in the cellular adaptation to high saline conditions^1^,^4^,^9^. Chen *et al.*^55^ showed that NO signalling induced salt resistance by increasing the K^+^/Na^+^ ratio and decreasing the Na^+^ content in *K. obovata.* Garcia-Mata *et al.*^59^ also found that NO initiated the abscisic acid-related signalling cascade that finally activated the guard cells of the K^+^ and Cl^-^ channels. Similarly, in this study, the NMT data showed that the low concentration of NaHS induced a net Na^+^ efflux in salt-treated barley seedling roots (Fig. 4b). Moreover, salt stress resulted in the activation of the PM H^+^-ATPase and the Na^+^/H^+^ antiporter. In our study, the gene transcription and protein levels of H^+^-ATPase and Na^+^/H^+^ antiporter increased significantly in the salt-treated barley seedling roots (Figs 6 and 8a–d), which might lead to Na^+^ transport through the PM and presumably further regulation of the cytosolic ion homeostasis. Additionally, we found that 100 μM NaHS evoked the protein expression of PM H^+^-ATPase and the transcriptional levels of *HvHA1* and *HvSOS1* genes in barley seedling roots (Figs 6a–c and 8a), which had close relationships with lower levels of cytoplasmic Na^+^.

Furthermore, the Na^+^ compartmentation into the vacuoles was an alternative solution to the challenge of salt stress^60^. This process depended on the protein expression and the activity of the vacuolar membrane H^+^-ATPase and Na^+^/H^+^ antiporters^5^. The H^+^-ATPase in the vacuolar membranes generated the proton gradient that was required for the activation of the Na^+^/H^+^ antiporter^61^. In this study, the NaHS greatly induced the transcriptional abundance of *HvVNHX2* and *HvVHA-β* genes in the salt-treated barley seedling roots (Fig. 8b,d). Moreover, H_2_S also significantly increased the protein expression level of vacuolar Na^+^/H^+^ antiporter (NHE1) (Fig. 6c,d). These results suggested that H_2_S might regulate the Na^+^ sequestration into the vacuoles, although the details of the mechanism require further investigation. The above results were in accordance with our previous study on the role of NO in the increased salt tolerance of mangroves^55^.

### A potential correlation between H_2_S and NO for plant tolerance

An endogenous signalling molecule, NO mediates the responses to multiple biotic and abiotic stresses, including salt, drought, and heat stress and disease resistance in plants^29^,^62^. Wang *et al.*^43^ reported that H_2_S increased the germination of seeds of *Medicago sativa* under saline conditions through a NO pathway. Similarly, in the present study, as shown in Fig. 10, a rapid generation of endogenous NO occurred in the salt-treated seedling roots after exposure to exogenous H_2_S, whereas in the barley seedling roots with cPTIO treatment, the H_2_S-induced NO production was blocked (Fig. 10), which was consistent with the results that were described by Wang *et al.*^43^. Furthermore, the application of the NO donor SNP mimicked the effects of H_2_S. For example, the NO functioned as a downstream signal to H_2_S to regulate the K^+^/Na^+^ balance and the K^+^ loss through an up-regulation of the gene expression of *HvAKT1* and *HvHAK4* in salt-induced barley seedling roots (Fig. 9e,f). Additionally, the NO and H_2_S remarkably increased the protein expression of PM H^+^-ATPase and the transcriptional levels of *HvHA1* and *HvSOS1* genes in barley seedling roots (Figs 7a,b and 9a,c), which might dramatically contribute to the maintenance of lower levels of cytoplast Na^+^. Furthermore, the NO and H_2_S significantly increased the transcriptional level of the *HvVNHX2* and *HvVHA-β* genes and increased the protein expression of the vacuolar Na^+^/H^+^ antiporter (NHE1) in salt-treated barley seedling roots (Figs 9b,d and 7c,d), which indicated that H_2_S and NO might function in the compartmentation of Na^+^ into the vacuoles. Based on these results, we concluded that H_2_S increased the salt tolerance through NO-regulated ion homeostasis, the protein and gene expression of the H^+^-ATPase and the Na^+^/H^+^ antiporter, and the activation of K channels in the barley seedling roots.

### Pathway of H_2_S-enhanced salt tolerance through NO-regulated ion homeostasis in salt-treated barley seedling roots

**Figure 11:**
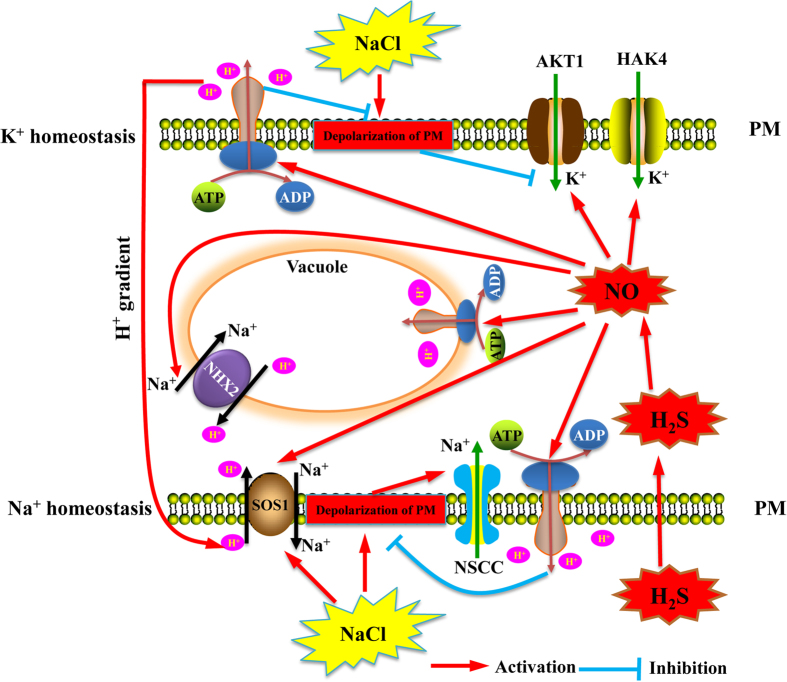
A schematic model for H_2_S enhances salt tolerance through NO-mediated maintenance of ion homeostasis in barley seedling roots.

Based on these results, we proposed a signalling transduction pathway through which H_2_S and NO mediated the K^+^/Na^+^ homeostasis in salt-treated barley seedling roots. As shown in Fig. 11, NaCl caused the depolarization of the PM, which led to a NSCCs-dependent over-influx of Na^+^ into the cytoplasm and the DA channels-mediated promotion of K^+^ efflux. Therefore, the excessive Na^+^ restricted the assimilation of K^+^ because of its competition for the sites of K^+^ uptake. Notably, H_2_S significantly simulated the production of NO under salt stress, which resulted in the activation of the PM H^+^-ATPase and the extrusion of H^+^ ions. The effect was multifaceted in that the activation the PM H^+^-ATPase was dependent on ion homeostasis (Fig. 11). On the one hand, the strengthened pumping of H^+^ could relieve the NaCl-induced depolarization of the PM and limit the efflux of K^+^ efflux. Whereas one the other hand, the increased extrusion of H^+^ by the H^+^-ATPase to ensure the proton gradient enabled the PM Na^+^/H^+^ antiporter (SOS1) to discharge Na^+^ from the cytoplasm. Moreover, the increase in H^+^ pumping by NO was also important for the sequestration of Na^+^ into the vacuoles by the vacuolar Na^+^/H^+^ antiporter, which would decrease the toxicity of Na^+^ for the plant cell. Additionally, the H_2_S-induced production of NO could reinforce the Na^+^ efflux outside the cytosol with the activation of the PM Na^+^/H^+^ antiporter system. Notably, the intracellular Na^+^ was involved in the suppression of the activities of AKT1 and HAK4, which suggested that the reduced cytoplasmic Na^+^ content via NO could promote the uptake of K^+^ by potential PM K^+^ carriers such as AKT1 and HAK4, leading to higher K^+^ concentrations and K^+^/Na^+^ ratios in the cytoplasm of salt-treated barley seedling roots.

Our results suggested that H_2_S and NO cooperated to improve salt tolerance by increasing the K^+^/Na^+^ ratio, by increasing the expression of the H^+^-ATPase and the Na^+^/H^+^ antiporter, and by increasing the activity of K channels in barley seedling roots. Over all, complex signalling networks exist in barley to increase the tolerance to salt stress, and different events may be required for plant development and tolerance to environmental stresses.

## Methods

### Plant growth and treatment

Uniform seeds of barley (*Hordeum vulgare* L.) were sterilized and then germinated according to the method of Chen *et al.*^26^. After germination, the seedlings were cultivated in a controlled growth chamber with a light/dark regime of 12/12 h, a temperature of 20/25 °C (night/day), a relative humidity of 75–85%, and a photon flux intensity (PFD) of 180 μmol m^−2^ s^−1^. The seedlings were grown for 3 days and were treated with various treatment solutions.

NaHS was used as an exogenous H_2_S donor^19^. Three-day-old seedlings of uniform size were chosen and separated into four groups for further treatment. In the first group, the seedlings were cultivated with 1/2 Hoagland’s solution that contained increasing concentrations of NaCl (0, 100, 200, 400 and 600 mM) at a pH of 6.0 for 24, 48, 72 and 96 h. In the second group, the seedlings were treated with solutions that contained different concentrations of NaHS (0, 50, 100, 200 and 500 μM) plus 150 mM NaCl at a pH of 6.0 for 48 h. In the third group, the seedlings were treated with 50 μM NaHS, 100 μM NaHS, 150 mM NaCl, 150 mM NaCl+50 μM NaHS and 150 mM NaCl+100 μM NaHS for 48 h. In the fourth group, an exogenous donor of NO (SNP, sodium nitroprusside), and a NO scavenger (cPTIO, 2-(4-carboxyphenyl)-4,4,5,5-tetramethylimidazoline-1-oxyl-3-oxide) were used^7^. The seedlings were grown for 3 days and then treated for 48 h as follow: (1) CK, Hoagland solution only without adding NaHS and NaCl; (2) Na, 150mM NaCl; (3) Na+S, 150 mM NaCl+100 μM NaHS; (4) Na+S+c, 150 mM NaCl+100 μM NaHS+200 μM cPTIO; (5) Na+N, 150 mM NaCl+100 μM SNP; and (6) Na+N+c, 150 mM NaCl+100 μM SNP+200 μM cPTIO. In this study, the NaHS, SNP and cPTIO solutions were prepared from higher concentration stock solutions (NaHS: 1 mM; SNP: 1 mM; and cPTIO: 1 mM) and then diluted to the lower concentration solutions of NaHS (50, 100, 200, 500 μM), SNP (100 μM) and cPTIO (200 μM) for our subsequent experiments.

### Measurements of root length, plant height, biomass and relative water content

The root length and plant height were measured with a ruler, and the biomass was measured with an analytical balance. The relative water content (RWC) was measured with the method of Yoo *et al.*^63^.

### Measurement of Na^+^ and K^+^ contents

The contents of Na^+^ and K^+^ were measured as described by Chen *et al.*^64^. After three rinses with distilled deionized water, the root apices and leaves were collected and dried at 65 °C for 48 h. The dried samples (50–100 mg) were incubated with 5 ml of concentrated HNO_3_ (65–68%) in digestion vessels, and then were digested in a microwave digestion system (CEM Inc., Mars-V, USA). Finally, the solutions were diluted to a specified volume with distilled deionized water and filtered through a 0.25 μm pore membrane filter. The Na^+^ and K^+^ contents were measured by inductively coupled plasma mass spectrometry (ICP-MS, PerkinElmer Inc., Elan DRC-e).

### Measurements of net Na^+^ and K^+^ fluxes

The Scanning Ion-selective Electrode Technique (SIET), as one of the Non-invasive Micro-test Techniques (NMT), is currently applied in plant research to monitor non-invasively outward or inward fluxes of specific ions and/or molecules from the cell without damaging the sample with a micro-electrode^34^,^65^. In this study, the net Na^+^ and K^+^ fluxes in the root tips of the barley seedlings were detected noninvasively using an SIET system (BIO-001A; Younger USA Sci. and Tech. Corp., Amherst, MA, USA), as described previously by Sun *et al.*^47^. The Na^+^ and K^+^ fluxes were recorded at approximately 1–2 seconds per point, which was required in case of the mechanical disturbance caused by the movement of electrode^4^. Additionally, the calibration of the ion-selective electrodes was performed before the flux measurements according to solutions that contained different concentrations of Na^+^ (0.5 mM, 0.9 mM and 5 mM) and K^+^ (0.05 mM, 0.1 mM and 0.5 mM). Fick’s law of diffusion was used to calculate the final Na^+^ and K^+^ fluxes as described by Xu *et al.*^66^. The net ion efflux or influx of the root was represented with negative or positive values, respectively, with at least six individual repeats. Each point in Figs 4 and 5 represents the mean ion flux of six individual roots. The data and image acquisition, preliminary processing, control of the positioned three-dimensional electrode, and stepper-motor-controlled fine focus of the microscope stage were performed using the Mage Flux software (http://www.youngerusa.com/mageflux or http://xuyue. net/mageflux)^47^,^67^.

### Steady-state SIET measurement of ion flux

The Na^+^ and K^+^ fluxes were measured using the SIET system in the roots of barley seedlings treated with 50 μM NaHS, 100 μM NaHS, 150 mM NaCl, 150 mM NaCl+50 μM NaHS and 150 mM NaCl+100 μM NaHS for 48 h. According to the methods of Sun *et al.*^47^, the roots were first washed with double-distilled water to avoid the effects of preloaded ions diffused from the surface of the high salinity-treated barley roots on the ion flux measurements, and then the roots were immediately incubated in the measuring solution (0.1 mM CaCl_2_, 0.1 mM KCl, 0.2 mM Na_2_SO_4_, 0.5 mM NaCl, and 0.3 mM MES, pH 6.0) to equilibrate for 30 min. Subsequently, the equilibrated samples were fixed at the bottom of 3 cm diameter plastic dishes that contained 2–3 ml of fresh measuring solution. The net Na^+^ and K^+^ fluxes were measured in constant conditions for 10 to 15 min to ensure that no fluctuations occurred.

### Transient K^+^ flux kinetics

For the transient K^+^ flux kinetics, the barley seedlings were separated into two groups for the following experiments. In the first group, the barley seedlings were pre-treated with 150 mM NaCl for 12 h. The roots were washed with double-distilled water and then were equilibrated in the measuring solution for 30 min. After a constant value for K^+^ was recorded for 6–7 min, the kinetics of the K^+^ fluxes was examined with a transient supply of 50 μM or 100 μM NaHS. A steady flux of K^+^ was recorded for at least 5 min, and the NaHS (50 μM or 100 μM) was then added, which was followed by the measurement of the net K^+^ flux for another 16–20 min. In the second group, the barley seedlings were pre-treated with different concentrations of NaHS (0, 50, 100 and 500 μM) for 30 min. Subsequently, the roots were washed with double-distilled water and then were equilibrated in the measuring solution for 30 min. After a steady flux of K^+^ was recorded for 6–7 min, the kinetics of the K^+^ fluxes was examined with the transient addition of 150 mM NaCl. A steady flux of K^+^ was recorded for at least 5 min before the addition of the NaCl. Then, the net K^+^ flux was measured for another 16–20 min after the addition of 150 mM NaCl. Because of the diffusion of the NaHS or the NaCl, all the experimental data were recorded after the first 2–3 min (in this study, the blank measurements, i.e., the without root condition, were performed to exclude the disturbance caused by the NaHS or NaCl additions on the K^+^ flux measurements). However, the net Na^+^ flux in these transient experiments was not determined because the measuring solution that contained high Na^+^ concentrations after salt stress for 12 h reduced the sensitivity of the Na^+^ selective electrode^47^.

### SDS-PAGE and western-blot analysis

0.5 g of barley seedlings root tips were used for proteins extraction as described by Chen *et al.*^26^. Protein concentrations were determined according to the method of Bradford^68^. Proteins (50 μg from each sample) were separated by SDS-PAGE using 12% (w/v) acrylamide gels and electrophoretically transferred to polyvinylidene difluoride (PVDF) membrane for 50 min for western-blot analysis^69^. The PVDF membrane was blocked overnight with Western Blocking Buffer (TIANGEN, China). Immunoblot analysis was performed using primary antibodies of plasma membrane H^+^-ATPase (1:1000, AS07260, Agrisera, Sweden) or vacuolar Na^+^/H^+^ antiporter (1:1000, AS09484, Agrisera, Sweden). Then blots were washed three times in PBST solution (50 mM Tris-HCl, pH 8.0, 0.05% Tween 20, 150 mM NaCl, v/v), followed by incubation with the secondary antibody (anti-rabbit IgG horse radish peroxidase conjugated, Abcam, U.K., 1:5000 dilution) for another 2 h at room temperature. β-actin (1:5000; Santa Cruz, California, USA) was used as an internal control. Finally, according to the manufacturer’s instructions, the blots were washed as above and developed with SuperSigmal West Pico Chemiluminescent Substrate (Pierce, USA). A CCD imager (FluorSMax, Bio-Rad, USA) was used to obtain images of the blots. The optical density value was calculated using the Quantity One software (Bio-Rad, Hercules, CA, USA). The comparative optical density value was used to determine the relative amount of protein expression.

### Total RNA extraction and gene expression analysis

To analyze the level of gene expression, total RNA samples were prepared from roots (0.3 g) using 2% polyvinylpyrrolidone and RNA purification reagent (Invitrogen Inc., CA, USA) according to the manusfacturer’s instructions. The RNA concentration was determined by measuring the optical density at 260 nm using an ultraviolet spectrophotometry (Cary 50, Varian, USA), while the integrity of RNA was tested by 1% agarose gel electrophoresis. Reverse transcription was performed using M-MLV reverse transcriptase (TaKaRa, Dalian, China) and the resulting product were used as template for qRT-PCR analysis, which was carried out by using Faststart Universal SYBR Green Master (ROX, Mannheim, Germany). The primers used for qRT-PCR are listed in Supplementary Table S1. To run qRT-PCR, three independent replicates of PM H^+^-ATPase (*HvHA*), vacuolar H^+^-ATPase subunit β (*HvVHA-β*), PM Na^+^/H^+^ antiporter (*HvSOS1*), vacuolar Na^+^/H^+^ antiporter (*HvVNHX2*), inward-rectifying potassium channel (*HvAKT1*) and high-affinity K^+^ uptake system (*HvHAK4*) dsDNA synthesis were performed using the Rotor-gene-6000 Real-Time PCR system (Corbett Research, Mortlake, Australia) with *Hvactin* as an internal control and the PCR conditions as described in Supplementary Table S2. The relative transcriptional abundance value of these genes was expressed as 2^-ΔΔCt^ by using the method of comparative threshold cycle (C_t_)^70^.

### Fluorescent imaging of endogenous NO

The endogenous NO was monitored using the highly specific fluorescent probe, 3-amino, 4-aminomethyl-2′,7′-difluorescein diacetate (DAF-FM DA, Calbiochem)^71^. The roots were washed with 20 mM HEPES buffer (pH 7.4) for 20 min, were incubated in 10 μM DAF-FM DA in HEPES buffer for another 30 min, and finally were washed in HEPES three times for 15 min. The root tips were imaged by a TCS-SP2 confocal laser scanning microscope (Leica Lasertechnik GmbH, Heidelberg, Germany; excitation at 488 nm, and emission at 500–530 nm). The experiments were repeated six times. All manipulations were performed in the darkness at 25 ± 1 °C.

### Measurements of endogenous NO contents

The endogenous NO contents were determined using the method of the Griess reagent as described by Zhou *et al.*^72^. The absorbance of the NO content was determined at 540 nm by comparison with a standard curve of NaNO_2_.

### Statistical analysis

Thirty seedlings were used for the measurements of the root length, plant height and biomass. For the measurements of the physiological and biochemical indexes, at least three replicates were used. The statistical analyses were performed with the general linear ANOVA model procedure of SPSS 14.0 (SPSS Inc., Chicago, IL,USA), and the results are expressed as the mean ± SE. The post hoc comparisons were tested using the Tukey test at the significance level of *P* < 0.05.

## Additional Information

**How to cite this article**: Chen, J. *et al.* Hydrogen sulfide enhances salt tolerance through nitric oxide-mediated maintenance of ion homeostasis in barley seedling roots. *Sci. Rep.* **5**, 12516; doi: 10.1038/srep12516 (2015).

## Supplementary Material

Supplementary Information
