# Supplementary material for: Hydrogen sulfide enhances salt tolerance through nitric oxide-mediated maintenance of ion homeostasis in barley seedling roots
Source: Sci Rep. 2015 Jul 27;5:12516. doi: 10.1038/srep12516 (PMC4515593; doi:10.1038/srep12516)

Title: Hydrogen sulfide enhances salt tolerance through nitric oxide-mediated maintenance of ion homeostasis in barley seedling roots

Running title: H_2S enhances salt tolerance in barley roots

Authors: Juan Chen[1,2], Wen-Hua Wang[3], Fei-Hua Wu[2,4], En-Ming He[3], Xiang Liu[2], Zhou-Ping Shangguan[1], and Hai-Lei Zheng [2,*]

Table S1 Sequences of forward and reverse primers were used in qRT-PCR for gene expression analysis in salt-treated barley seedling roots.

Gene name	Forward primer sequence (5' to 3')	Reverse primer sequence (5' to 3')
HvHA	TCATCCACAGGACCGACTTCTTCACA	AAGGCGATAACCAAGAGGAAACCAG
HvVHA-β	AGGTTGGAGCAAAGCAAGCACGCAG	ATAATACGCTCAATAGTGGGGTCAT
HvSOS1	GAAGGCATTCTACTGACCGTGAG	ATGCGACATCACAATACCACAGA
HvVNHX2	TTCGTATTCCCTTTATCTTACCTATCC	GCATTAACTCGCACGGCAGTATGAC
HvAKT1	TCGCAATATCTGTTTCTCAATCTGG	GAAACTAAAATGTAGAAGTCAGTGGGTG
HvHAK4	TTACAAGTGCATCTGTCAGGCTAG	GTCCTGGGATGGAGTCATAAAAAC
Hvactin	GGGCAGAAGGATGCTTATGTTGGTG	TCCATGTCATCCCAGTTGCTTACGA

Table S2 Procedures of dsDNA synthesis were used in qRT-PCR for gene expression analysis in in salt-treated barley seedling roots.

Gene name	Procedures of dsDNA systhesis
HvHA	10 min at 95 °C and 40 cycles of 95 °C for 30 s, 54 °C for 30 s and 72 °C for 20 s
HvVHA-β	10 min at 95 °C and 40 cycles of 95 °C for 30 s, 52 °C for 30 s and 72 °C for 25 s
HvSOS1	10 min at 95 °C and 40 cycles of 95 °C for 30 s, 57 °C for 30 s and 72 °C for 20 s
HvVNHX2	10 min at 95 °C and 40 cycles of 95 °C for 30 s, 53 °C for 30 s and 72 °C for 20 s
HvAKT1	10 min at 95 °C and 40 cycles of 95 °C for 30 s, 52 °C for 30 s and 72 °C for 20 s
HvHAK4	10 min at 95 °C and 40 cycles of 95 °C for 30 s, 53 °C for 30 s and 72 °C for 20 s

Figure S1 The symptoms of barley seedlings after 96 h of treatment with different concentrations of NaCl (0, 100, 200, 400 and 600 mM). Each value is the mean ± SE (n = 30).

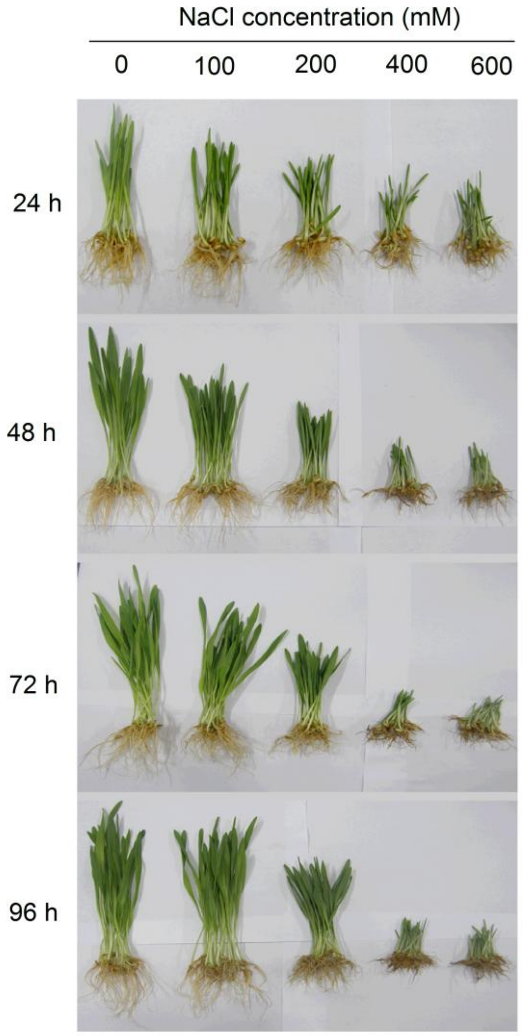

Supplement: Supplementary Information [file srep12516-s1.pdf]
